# The effect of ivermectin^®^ on fertility, fecundity and mortality of *Anopheles arabiensis* fed on treated men in Ethiopia

**DOI:** 10.1186/s12936-019-2988-3

**Published:** 2019-11-08

**Authors:** Wondemeneh Mekuriaw, Meshesha Balkew, Louisa A. Messenger, Delenasaw Yewhalaw, Adugna Woyessa, Fekadu Massebo

**Affiliations:** 1 Ethiopian Public Health Institute, Addis Ababa, Ethiopia; 2 Abt Associates, PMI Vectorlink Project in Ethiopia, Addis Ababa, Ethiopia; 3 Department of Disease Control, London School of Hygiene and Tropical Medicine, London, UK; 4 Tropical and Infectious Disease Research Center, Jimma University, Jimma, Ethiopia; 5 Department of Medical Laboratory Sciences and Pathology, College of Health Sciences, Jimma University, Jimma, Ethiopia; 6 Department of Biology, Arba Minch University, Arba Minch, Ethiopia

**Keywords:** *Anopheles arabiensis*, Fecundity, Fertility, Ivermectin, Mortality

## Abstract

**Background:**

Insecticide resistance is a growing threat to malaria vector control. Ivermectin, either administered to humans or animals, may represent an alternate strategy to reduce resistant mosquito populations. The aim of this study was to assess the residual or delayed effect of administering a single oral dose of ivermectin to humans on the survival, fecundity and fertility of *Anopheles arabiensis* in Ethiopia.

**Methods:**

Six male volunteers aged 25–40 years (weight range 64–72 kg) were recruited; four of them received a recommended single oral dose of 12 mg ivermectin and the other two individuals were untreated controls. A fully susceptible insectary colony of *An. arabiensis* was fed on treated and control participants at 1, 4, 7, 10 and 13 days post ivermectin-administration. Daily mosquito mortality was recorded for 5 days. *An. arabiensis* fecundity and fertility were measured from day 7 post treatment, by dissection to examine the number of eggs per mosquito, and by observing larval hatching rates, respectively.

**Results:**

Ivermectin treatment induced significantly higher *An. arabiensis* mortality on days 1 and 4, compared to untreated controls (*p *= 0.02 and *p *< 0.001, respectively). However, this effect had declined by day 7, with no significant difference in mortality between treated and control groups (*p *= 0.06). The mean survival time of mosquitoes fed on day 1 was 2.1 days, while those fed on day 4 survived 4.0 days. Mosquitoes fed on the treatment group at day 7 and 10 produced significantly lower numbers of eggs compared to the untreated controls (*p *< 0.001 and *p *= 0.04, respectively). *An. arabiensis* fed on day 7 on treated men also had lower larval hatching rates than mosquitoes fed on days 10 and 13 (*p *= 0.003 and *p *= 0.001, respectively).

**Conclusion:**

A single oral dose of ivermectin given to humans can induce mortality and reduce survivorship of *An. arabiensis* for 7 days after treatment. Ivermectin also had a delayed effect on fecundity of *An. arabiensis* that took bloodmeals from treated individuals on day 7 and 10. Additional studies are warranted using wild, insecticide-resistant mosquito populations, to confirm findings and a phase III evaluation among community members in Ethiopia is needed to determine the impact of ivermectin on malaria transmission.

## Background

Malaria is a disease transmitted by female *Anopheles* mosquitoes, caused by protozoan parasites of the genus *Plasmodium*. The disease predominately occurs in tropical and sub-tropical regions and remains a major public health problem. Most malaria cases in 2017 occurred in the World Health Organization (WHO) African Region (92%), followed by the WHO South-East Asia Region (5%) and the WHO Eastern Mediterranean Region (2%). Of the 87 countries reporting indigenous malaria cases in 2017, 15 countries (all in sub-Saharan Africa) and India carried 80% of the global malaria burden. Malaria is estimated to have decreased by 20% in 20 countries, mainly due to the wide use of vector control interventions, and increased with a similar magnitude in another 20 countries between 2016 and 2017 [1].

Current malaria vector control programs mainly rely on the use of chemical insecticides from five classes [2]. However, the development of insecticide resistance [3, 4] and behavioural changes, including early and outdoor biting activities [5], may jeopardize the effectiveness of malaria vector control interventions. In Ethiopia, vector behavioural changes, such as behavioural avoidance, feeding on animals, resting outdoors away from indoor treated surfaces, and feeding upon humans when they are not protected, all contribute to sustaining residual malaria transmission [6]. The zoophagic tendency of *Anopheles arabiensis* [7] presents an opportunity to control this species by treating cattle with ivermectin and it is an important option to target zoophagic mosquitoes.

To achieve malaria elimination before 2030, as set by the WHO [8], innovation of vector control tools to counteract the emergence of drug and insecticide resistance is fundamental [9]. For this reason, ivermectin is receiving more attention as a potential tool to be used for malaria control [9–11]. This drug has been used since the 1980s for animal health to control parasitic diseases, including cattle onchocerciasis [12, 13], lymphatic filariasis [14], and scabies and lice [15, 16]. Evidence for the efficacy of ivermectin to reduce *Anopheles* survivorship and *Plasmodium* sporozoite rate is growing. Mass drug administration of ivermectin in southeastern Senegal for onchocerciasis and lymphatic filariasis dramatically affected the density of malaria vectors and reduced the proportion of *Plasmodium falciparum* infectious mosquitoes [17]. Furthermore, the survivorship of *Anopheles gambiae* sensu stricto (s.s.) that ingested blood of humans treated with 200 μg/kg ivermectin was reduced significantly after 1 day of treatment [18]; however, this effect was not apparent 14 days post-ingestion. This might be because ivermectin and/or its metabolites are removed from plasma over 12 days after treatment [19]. Even a single dose of ivermectin in small concentrations, can have a deleterious effect on mosquitoes before they become infectious and can reduce survival [18, 20]; ivermectin-exposed mosquitoes are less likely to transmit *Plasmodium* parasites due to a shift in *Anopheles* populations to younger mosquitoes [10].

The implementation of ivermectin could contribute to insecticide resistance management. The occurrence of cross-resistance to ivermectin from currently used insecticides is less likely as its mechanism of action (inhibition of glutamate-gated chloride channels) is different [21]. However metabolic resistance could still affect the impact of ivermectin on mosquito mortality [22]. Treatment of cattle or humans with ivermectin, may represent a viable complementary vector control strategy. The aim of this study was to assess the effect of administering a single oral dose of ivermectin to humans on mortality, fecundity and fertility of a laboratory colony of *An. arabiensis* in Ethiopia.

## Methods

### Study area, design and participants

This study was conducted from July 2017 to October 2017 at the Tropical and Infectious Diseases Research Centre, Jimma University in Sekoru, Oromia region (7.922305° N, 37.395320° E). The research centre is 246 km South-West of Addis Ababa, situated at an altitude of 1684 m above sea level. Six volunteer males aged 25–40 years, weighing between 64 and 72 kg were recruited from Sekoru village, after receiving informed consent. The dosage of ivermectin given to volunteer range from 166.7 to 187.5 µg/kg. The volunteers were assigned randomly to either treatment or control groups using a lottery method. Four of them received a recommended single oral dose of 12 mg ivermectin for these weights and the other two individuals did not receive the drug (untreated controls). The entomology technicians (providing mosquitoes for feeding and performing mosquito dissections) were blinded to the treatment and control groups. The drug was obtained from the Ministry of Health, donated by the Mectizan donation program for onchocerciasis elimination in Ethiopia.

### Experimental procedure

#### Mosquito rearing in the laboratory

An insecticide-susceptible colony of *An. arabiensis* (Debre Zeit: DZ) [23], reared under standard conditions of 27 ± 2 °C, 70 ± 10% relative humidity and 12 h/12 h day/night cycles, was used for this experiment. Larvae were reared in plastic trays in distilled water and were provided a diet of ground Tetramin^®^ fish food. Pupae were collected in cups and placed in 30 × 30 × 30 cm cages. Emerging adults were provided with 10% sugar solution for 3–5 days.

#### Mosquito feeding

**Fig. 1:**
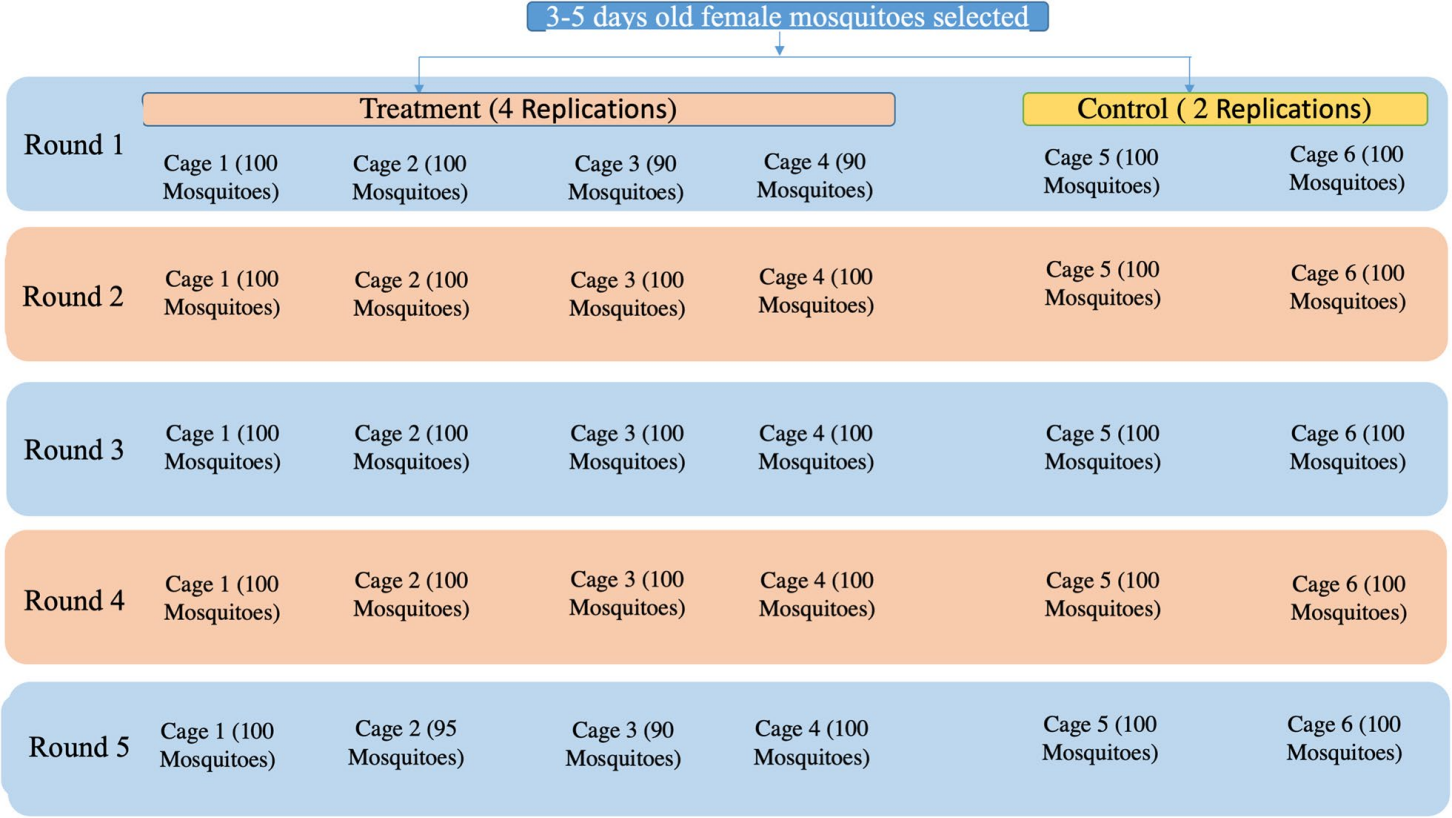
Flow chart of the mosquito feeding procedures

Prior to human feeding, female 3–5 day-old, *An. arabiensis* were starved of sugar solution for 4 h. Twenty-four hours after ivermectin ingestion, human volunteers exposed their right arm to 90–100 starved female *An. arabiensis* inside cages. A total of six cages (four for the ivermectin group, two for the untreated control) were used. Mosquitoes were allowed to feed for 30 min. Unfed and fully un-engorged mosquitoes were removed from the cages using mouth aspirators. Then, fully fed mosquitoes were maintained on 10% sugar solution for 5 days. Mosquito mortality was recorded every 24 h for those 5 days. The feeding experiment was repeated at days 4, 7, 10 and 13 post-ivermectin treatment using different batches of mosquitoes, following the same procedure (Fig. 1).

#### Mortality estimation of *Anopheles arabiensis*

Daily mortality of mosquitoes was monitored for 5 days after feeding for both treatment and control groups in each round. Dead mosquitoes were recorded and removed from the cages every day. New batches of *An. arabiensis* were used in each replicate. Mosquitoes were considered dead if they were lying on the bottom of the cage and unable to move. If a mosquito was unable to fly yet it was able to stand on its legs, it was considered alive [24].

#### Fecundity estimation of *An. arabiensis*

Mosquito fecundity was measured starting day 7 post treatment because of the high mortality rate in the early days of follow-up. Mosquitoes were offered a blood meal from a volunteer on day 7, 10 and 13. To determine fecundity, 80 gravid mosquitoes from treatment groups and 40 from the control group were dissected on the fourth day after their blood meal using a dissecting microscope in round 3, 4 and 5. For dissection, female mosquitoes were anaesthetized using chloroform, placed on a clean microscope slide in a drop of distilled water. The thorax of a mosquito was gently grasped by a dissecting needle and the last two abdominal segments were gently pulled away using another dissecting needle to count the number of eggs (partially-formed eggs were also counted) in both ovaries.

#### Fertility estimation of *An. arabiensis*

In each round an additional 80 gravid *An. arabiensis* from the treatment group and 40 from the control group were gently transferred to individual 1.5 ml Eppendorf tubes, containing a moist triangular piece of Whatman No. 1 filter paper with air holes in the cap and base, using a mouth aspirator. Laid eggs from the experimental and control groups were reared in separate plastic cups filled with distilled water. Hatched larvae were supplied with fish food (TetraMin^®^) daily in larval pans. The number of newly emerged larvae were recorded. Eggs that could not develop into larvae up to day 7 were considered infertile.

#### Data analysis

Data were recorded in appropriately designed forms, entered into Microsoft excel for data cleaning and exported to SPSS version 16 and R version 3.4.4 software for analysis. Analysis of variance were used to compare mean mortality, fecundity and fertility of mosquitoes between treatment and control groups as well as among individual experimental group replicates. Descriptive statistics were used to calculate feeding rates of mosquitoes and hatching rates of eggs. Survivorship of mosquitoes was analysed using Kaplan–Meier survival curves. Mortality of mosquitoes was standardized and pooled prior to data analysis.

## Results

### Effect of ivermectin on *Anopheles arabiensis* mortality

**Fig. 2:**
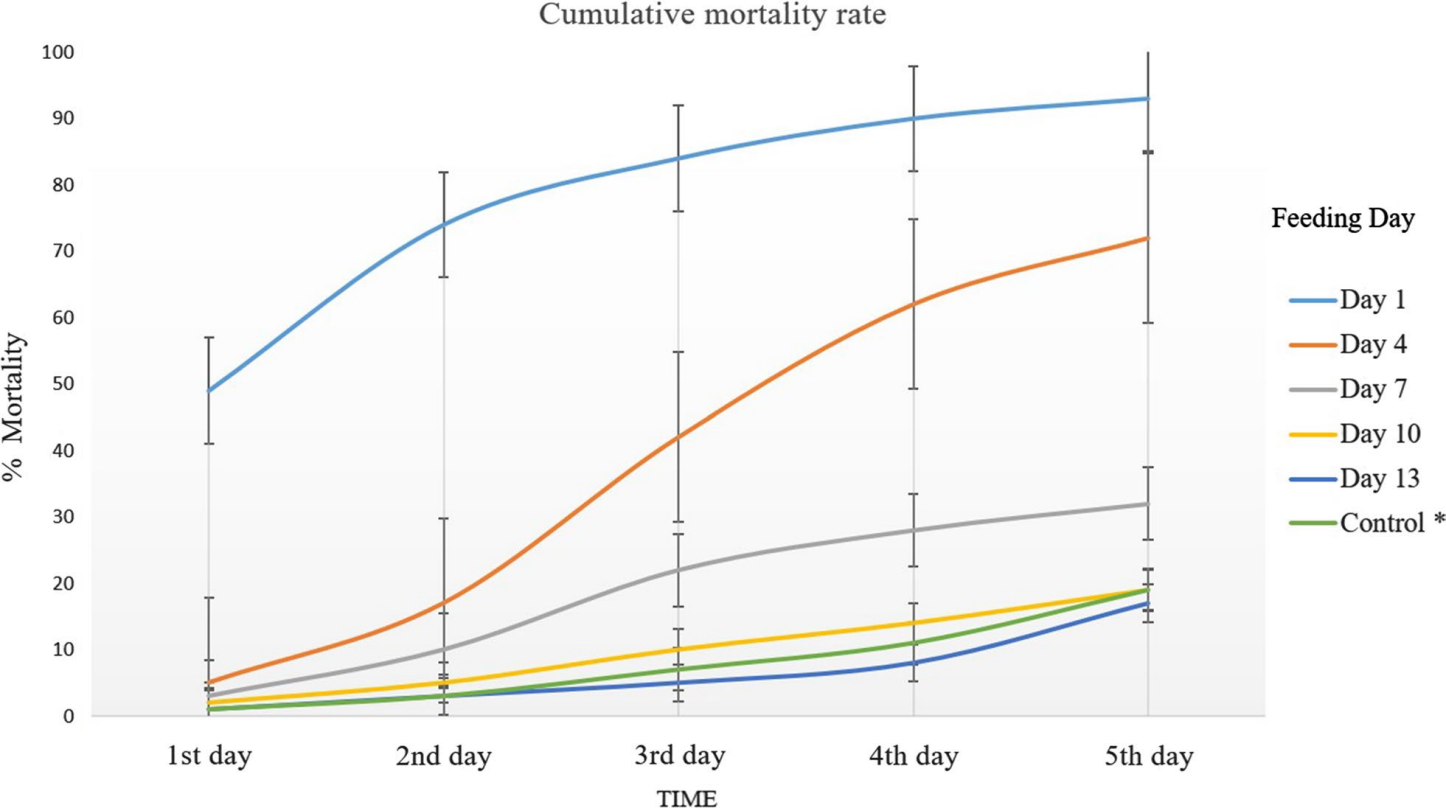
The cumulative mortality rate of *An. arabiensis* during 5-days of follow-up at different feedings days, post-ivermectin treatment, July 2017, Sekoru. *Five rounds of mean mortality of mosquitoes in the control group

The number of *An. arabiensis* exposed to feed on humans were 2965 and out of this 2465 mosquitoes became fully engorged. The feeding rate of mosquitoes was 83.1% and an average of 493 mosquitoes were used per replicate. There were no significant differences in mosquito feeding rates between treatment and control groups (82.5% and 84.5% average feeding rates, respectively *p *= 0.355). Overall feeding rates of mosquitoes at different feeding days are shown in Table 1. The mean mortality rate during the 5-day follow-up was high on day 1, 4 and 7 post-treatment compared to the controls. The mean daily mortality of mosquitoes fed on days 10 and 13 was similar to the control mortality (Table 2 and Fig. 2).

**Table 1:** Feeding rates of mosquitoes at different feeding times

Feeding day	Treatment				Control	
	Replicate 1	Replicate 2	Replicate 3	Replicate 4	Replicate 1	Replicate 2
1DAT	74.00	80.00	81.11	86.67	83.00	77.00
4DAT	80.00	91.00	73.00	81.00	86.00	82.00
7DAT	87.00	86.00	79.00	82.00	94.00	83.00
10DAT	90.00	86.00	79.00	77.00	86.00	85.00
13DAT	87.00	83.16	94.44	72.00	92.00	78.00

*DAT* days after treatment

**Table 2:** Mean daily mortality of *An. arabiensis* fed on treated or control volunteers at different feeding days after ivermectin administration and follow-up for five consecutive days

Feeding day	Exposure	Mosquito, N	Mean daily mortality	95% CI for mean		*P* value
				Lower bound	Upper bound	
1DAT	Treatment	76	13.83	7.84	19.81	0.02
	Control	80	3.70	1.82	5.59	
4DAT	Treatment	81	11.19	8.52	13.87	0.00
	Control	84	3.46	1.35	5.57	
7DAT	Treatment	84	4.74	3.57	5.91	0.06
	Control	89	2.85	0.99	4.70	
10DAT	Treatment	83	2.76	2.26	3.25	0.38
	Control	86	2.33	1.23	3.43	
13DAT	Treatment	81	2.52	1.45	3.59	0.80
	Control	85	2.73	1.33	4.14	

Feeding day: the day when *An. arabiensis* mosquitoes took a bloodmeal from ivermectin treated or control volunteers after drug administration

*CI* confidence interval, *DAT* days after treatment, *N* number of *An. arabiensis*

The mean number of dead *An. arabiensis*, after feeding on ivermectin-treated individuals on day 1 post administration, was 13.8; this was significantly higher than mortality after feeding on control individuals (3.7 dead; *p *= 0.02), during 5 days of follow-up (Table 2). Similarly, the mean mortality of *An. arabiensis* fed on treated volunteers 4 days post ivermectin treatment was also significantly different from controls (*p *< 0.001). There was no difference in mosquito mortality between treatment and control groups from day 7 post ivermectin administration onwards (Table 2).

**Fig. 3:**
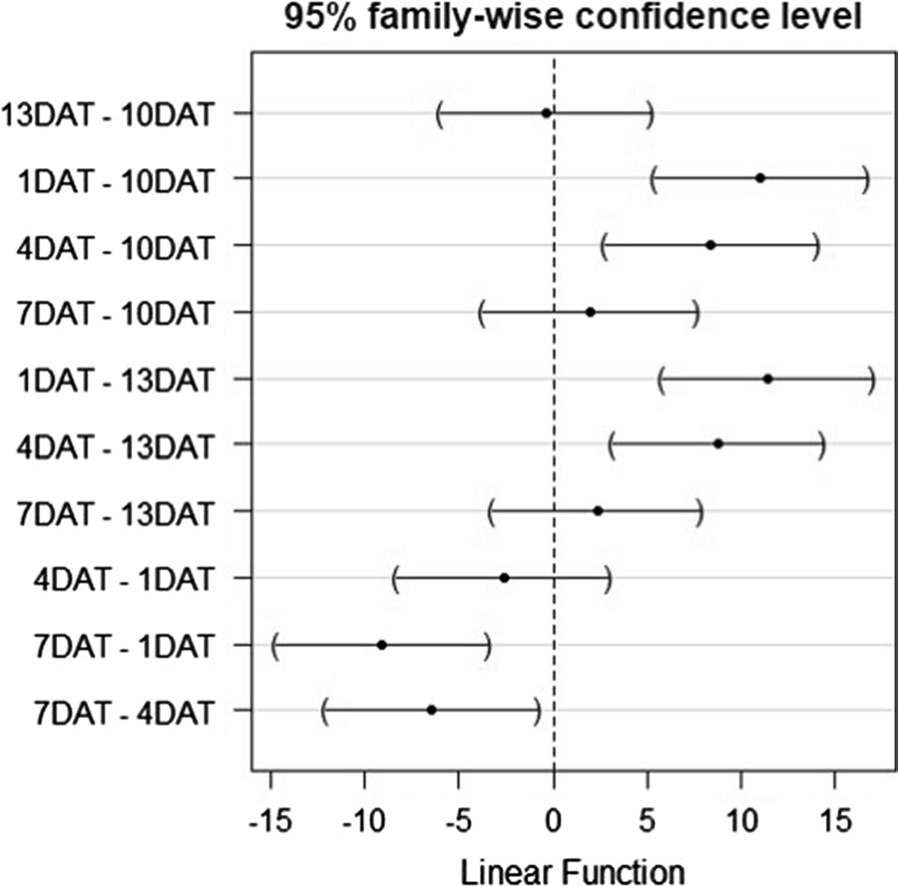
Pairwise comparisons of mean daily mortality of *An. arabiensis* at different feeding days after ivermectin administration. *DAT* days after treatment. Mortality was not significantly different between feeding days, where mean difference confidence intervals cross the middle construction line

The effect of ivermectin on mean 5-day mortality of *An. arabiensis* between different feeding intervals varied among treatment groups and controls, according to a one-way ANOVA and a post hoc multiple comparisons test (Fig. 3). Mean daily mortality of *An. arabiensis* fed on ivermectin treated volunteers on day 1 and day 4 were not significantly different (*p *= 0.73) (Fig. 3). By comparison, mean daily mortality of *An. arabiensis* fed on day 1 was significantly higher than those feed on days 7, 10 and 13 (*p *< 0.001). In addition, mean daily mortality of *An. arabiensis* fed on day 4 post treatment, was significantly different from day 7 (*p *= 0.011, 95% CI 0.9824–11.92), day 10 (*p *< 0.001, 95% CI 2.96–13.90) and day 13 (*p *< 0.001, 95% CI 3.20–14.15). However, mean daily mortality rates of *An. arabiensis* fed on ivermectin-treated volunteers on days 7, 10 and 13 were not statistically different (*p *= 0.900, 95% CI 3.49–7.45).

### Effect of ivermectin on *An. arabiensis* fecundity

The effect of ivermectin on *An. arabiensis* fecundity on days 1 and 4 after treatment was not assessed due to high mortality of *An. arabiensis* during this period. The mean number of eggs laid by *An. arabiensis*, which fed on treated volunteers on day 7, was 42.24 ± 6.60, compared to 110.05 ± 4.81 in the control group (*p *< 0.001). The mean number of eggs laid by *An. arabiensis*, which took blood meals from treated volunteers on day 10 post ivermectin administration was also significantly different compared to the control group (*p *= 0.04). However, ivermectin did not have a significant effect on fecundity of *An. arabiensis* after day 13 post drug administration (*p *= 0.34) (Table 3).

**Table 3:** Mean number of eggs per mosquito fed on ivermectin treated and non-treated volunteers at different feeding times

Feeding day	Exposure	Mosquito N	Mean no. eggs/ovary	95% CI for mean		P-value
				Lower bound	Upper bound	
7DAT	Treatment	80	42.24	31.74	52.73	< 0.001
	Control	40	110.05	66.85	153.25	
10DAT	Treatment	80	94.99	84.98	105	0.04
	Control	40	112.35	46.91	177.79	
13DAT	Treatment	80	114.6	110.31	118.89	0.34
	Control	40	110.85	52.4	169.3	

Feeding day: the day when *An. arabiensis* mosquitoes took a bloodmeal from ivermectin treated or control volunteers after drug administration

*SD* standard deviation, *SE* standard error, *CI* confidence interval, *N* number of gravid *An. arabiensis*

The effect of ivermectin on fecundity of *An. arabiensis* were compared between days 7, 10 and 13 after treatment. The mean number of eggs laid by *An. arabiensis*, which fed on treated volunteers on day 7 was significantly different compared to day 10 (mean difference: 52.7; 95% CI 41.9–63.9; *p *< 0.001) and day 13 (mean difference: 72.3; CI 61.2–83.5; *p *< 0.001). There was also a significant difference in mosquito fecundity between days 10 and 13 (mean difference: 19.6; 95% CI 8.5–30.7; *p *< 0.001).

### Effect of ivermectin on *An. arabiensis* fertility

The effect of ivermectin on *An. arabiensis* fertility on days 1 and 4 post ivermectin treatment was also not assessed. Mean hatching rates of eggs, laid from mosquitoes fed on treated volunteers compared to the control group, were 73.8% vs. 91.3% on day 7, 88.6% vs. 91.9% on day 10 and 90.5% vs. 92.5% on day 13. Significant differences in hatching rates between *An. arabiensis*, fed on treated vs control individuals, were observed on day 7 (*p *= 0.03) but not days 10 and 13 (*p *= 0.07 and *p *= 0.34, respectively).

Mean hatching rate of eggs from *An. arabiensis*, which took a blood meal on day 7 after ivermectin administration, was significantly lower compared to day 10 (mean difference: 14%; 95% CI 6.8–23.45; *p *= 0.03) and day 13 (mean difference: 16%; 95% CI 8–25.3; *p *< 0.001). However, the effect of ivermectin on fertility was not significant between days 10 and 13 post treatment (mean difference: 1.9%; 95% CI 6.8–10.6; *p *= 0.82) (Table 4).

**Table 4:** Mean hatching rate of eggs from *An. arabiensis* mosquitoes fed on ivermectin treated and non-treated volunteers at different feeding days

Feeding day	Exposure	Mean no. of eggs laid	% mean hatching	95% CI for mean		P-value
				Lower bound	Upper bound	
7DAT	Treatment	44	73.85	62.37	85.33	0.03
	Control	110	91.29	78.73	103.85	
10DAT	Treatment	97	88.62	86.15	91.09	0.07
	Control	119	91.9	77.63	106.17	
13DAT	Treatment	116	90.53	87.5	93.56	0.34
	Control	122	92.51	90.82	94.2	

*SD* standard deviation, *SE* standard error, *CI* confidence interval

### Effect of ivermectin on *An. arabiensis* survival

The effect of ivermectin on *An. arabiensis* survival was assessed for five consecutive days post feeding on treated or control participants. All live mosquitoes on day 5 were considered as censored.

The mean survival time of *An. arabiensis*, which blood fed from ivermectin treated volunteers on day 1 post treatment was 2.1, compared to 5.5 in the control group (*p *< 0.001) (Table 5). Statistically significant differences in mean survival time of exposed *An. arabiensis*, compared to control mosquitoes, were also observed on day 4 (mean survival days: 4.02, 95% CI 3.69–4.36; *p *< 0.001) and day 7 (mean survival days: 5.06; 95% CI 4.73–5.39; *p *= 0.01), post treatment. Otherwise no statistically significant difference was demonstrated for days 10 and 13 (Table 5).

**Table 5:** Mean survival time of mosquitoes during 5-days of follow-up at different feeding days

Feeding day	Exposure	Mean survival time (days)	95% CI		X^2^	P-value
			Lower bound	Upper bound		
1DAT	Treatment	2.13	1.80	2.46	117.1	< 0.001
	Control	5.53	5.30	5.75		
4DAT	Treatment	4.02	3.69	4.36	49.9	< 0.001
	Control	5.60	5.39	5.80		
7DAT	Treatment	5.06	4.73	5.39	6.1	0.01
	Control	5.66	5.47	5.85		
10DAT	Treatment	5.47	5.20	5.74	0.0	0.87
	Control	5.56	5.34	5.77		
13DAT	Treatment	5.63	5.41	5.84	0.02	0.89
	Control	5.65	5.45	5.85		

**Fig. 4:**
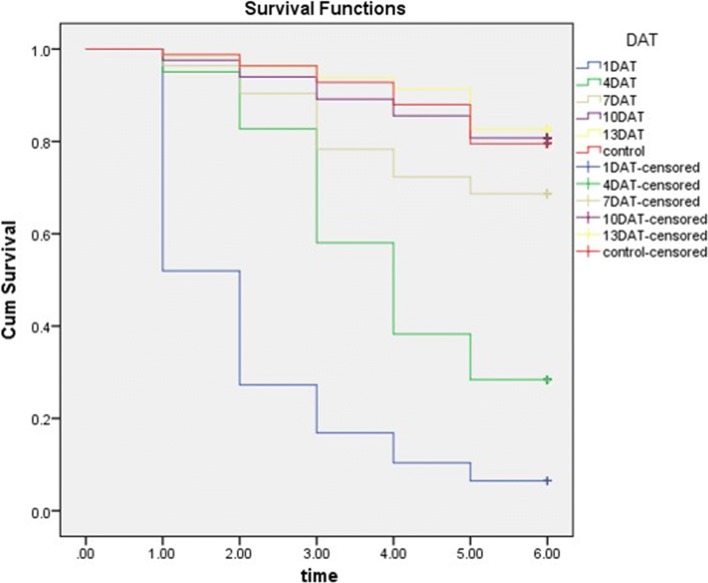
Kaplan–Meier survival curve of *An. arabiensis* during 5 days of follow-up after different feeding days post ivermectin treatment

As shown in Fig. 4, the majority of *An. arabiensis* that fed on treated volunteers on day 1 post ivermectin administration died within 2 days. By comparison, most *An. arabiensis*, which took a blood meal from treated volunteers on day 4 post-treatment, died within 4 days.

## Discussion

In this study, a single oral dose of ivermectin induced *An. arabiensis* mortality and reduced fecundity and fertility after feeding on treated men, compared to controls. Previous studies conducted on the effects of ivermectin on different disease vectors documented that ivermectin reduced the survivorship of *Anopheles stephensi*, *Aedes aegypti*, *Culex pipiens* and *Culex quinquefasciatus* [25]. The majority of tested mosquitoes, which fed 1 day post treatment died within 2 days and 93% mortality was recorded on day 5 post-ingestion. In agreement with the present study, results from Kenya showed that nearly half of blood fed *An. gambiae* from the ivermectin group died on the second day and 90% died on day 6, compared to 9% mortality in the control group on day 6 [26]. In addition, a dose of 300 μg/kg ivermectin per day given for 3 days to malaria patients can reduce mosquito survival for at least 28 days after treatment [27]. Multiple studies support the observation that mass drug administration of ivermectin reduces the survivorship of mosquitoes [28, 29]. In West Africa, following ivermectin mass drug administration (MDA), survival of *An. gambiae* declined by more than 33% for 6 days, with reductions of sporozoites by 77% in the following 2 weeks; and a reduction in parity rate was also observed [30]. Ivermectin-containing blood meals have also been shown to reduce the survivorship of principal malaria vectors in different parts of the world [18, 20, 28, 29, 31–33].

The present study revealed that mosquito survival was significantly reduced after the ingestion of ivermectin from treated humans on 1 and 4 days after treatment. Mortality of mosquitoes started to decrease from day 7. Therefore, day 7 could be the re-dosing time of ivermectin in this context. This is one pharmacological strategy recommended by the WHO to increase the efficacy of ivermectin [9]. However, this is hard to do logistically in the community unless a longer lasting formulation of ivermectin is developed in the future. The death rate of mosquitoes on day 10 and 13 day after treatment were similar to the control group, which may be attributed to the pharmacokinetics of ivermectin in the human body. Since the concentration of ivermectin and/or its metabolites are excreted in faeces every day, the residual concentration found in the plasma is also reduced day to day [16, 34].

The current study revealed that the number of eggs observed in the mosquitoes’ ovaries was reduced after the ingestion of ivermectin on 7 and 10 days after treatment. Studies conducted on *Anopheles aquasalis* documented a similar effect of ivermectin on mosquito fecundity [35]. In addition to this, a study by Gardner et al. [31] showed a reduction in fecundity of *Anopheles quadrimaculatus*, exposed to 24 µg/kg ivermectin. These and the current study findings indicate that ivermectin can reduce mosquito density, prior to completion of the gonotrophic cycle [36].

Ivermectin also impacted mosquito fertility at day 7 post-treatment, in agreement with previous studies done on cattle, which also showed reduced fertility [29]. The effect of ivermectin on fertility of *Ae. aegypti, Aedes albopictus*, and *Cx. quinquefasciatus* has been reported [37]. Furthermore, Gardner et al. [31] also showed the effect of 24 µg/kg ivermectin on hatching rate of *An. quadrimaculatus* mosquitoes. All of these physiological effects contribute to the reduction of vector population density.

This study has several strengths and limitations. The study was conducted using susceptible colony mosquitoes and, therefore, these findings cannot yet be directly extrapolated to wild mosquito populations. Additional studies, in areas characterized by different insecticide resistance intensities and underlying mechanisms are warranted to validate these phenomena. However, the study showed the delayed effect of a single oral dose of ivermectin used to treat onchocerciasis on the mortality, fertility and fecundity of mosquitoes, which is considered as the strength of the study.

## Conclusions

A single oral dose of ivermectin provided to humans can induce mortality and reduce survivorship of *An. arabiensis* for 7 days after treatment. It also had a delayed effect on fecundity of *An. arabiensis* that took blood meal from treated individuals on day 7 and 10 after ivermectin administration. Moreover, a delayed effect on fertility was observed when *An. arabiensis* took blood meal from treated volunteers on 7 days after treatment. Together these effects demonstrated the potential for ivermectin to reduce *An. arabiensis* population densities.

## Recommendations

The effect of ivermectin on wild population survival, fecundity, and fertility must be studied before public health use. In addition, future studies are needed to investigate the delayed effects of ivermectin on survival, fecundity, and fertility of insecticide resistant mosquito populations in Ethiopia and in other malaria endemic countries.

## Data Availability

Not applicable.

## Abbreviations

DAT: days after treatment
EPHI: Ethiopian Public Health Institute
IRS: indoor residual spraying
LLINs: long-lasting insecticidal nets
MDA: Mass Drug Administration
PMI: President’s Malaria Initiative
RH: relative humidity
WHO: World Health Organization

## References

[1] WHO. Recommended insecticides for indoor residual spraying against malaria vectors. Geneva: World Health Organization; 2018. https://www.who.int/neglected_diseases/vector_ecology/vector-control/Insecticides_IRS_22_September_2018.pdf?ua=1. Accessed 17 Jun 2019.

[2] WHO (2018). World malaria report.

[3] Christian R, Koekemoer L, Fettene M, Olana D, Coetzee M (2013). Insecticide resistance in *Anopheles arabiensis* from Ethiopia. African Entomol..

[4] FY 2017 Ethiopian Malaria Operation Plan. 2017. https://www.pmi.gov/resource-library/mops/fy-2017. Accessed 20 Apr 2019.

[5] Kenea O, Balkew M, Tekie H, Gebre-Michael T, Deressa W, Loha E (2016). Human-biting activities of Anopheles species in south-central Ethiopia. Parasit Vectors..

[6] WHO (2014). Control of residual malaria parasit transmission.

[7] Massebo F, Balkew M, Gebre-Michael T, Lindtjørn B (2015). Zoophagic behaviour of anopheline mosquitoes in southwest Ethiopia: opportunity for malaria vector control. Parasit Vectors..

[8] WHO (2015). Global technical strategy for malaria 2016–2030.

[9] WHO (2016). Ivermectin for malaria transmission control: technical consultation meeting.

[10] Chaccour CJ, Kobylinski KC, Bassat Q, Bousema T, Drakeley C, Alonso P (2013). Ivermectin to reduce malaria transmission: a research agenda for a promising new tool for elimination. Malar J..

[11] Chaccour CJ, Rabinovich NR, Slater H, Canavati SE, Bousema T, Lacerda M (2015). Establishment of the Ivermectin Research for Malaria Elimination Network: updating the research agenda. Malar J..

[12] Campbell WC (2016). Ivermectin: a reflection on simplicity (Nobel lecture). Angew Chem Int Ed Engl.

[13] Thylefors B (2008). The Mectizan donation program (MDP). Ann Trop Med Parasitol.

[14] Ottesen EA, Hooper PJ, Bradley M, Biswas G (2008). The global programme to eliminate lymphatic filariasis: health impact after 8 years. PLoS Negl Trop Dis..

[15] Chosidow O, Giraudeau B, Cottrell J, Izri A, Hofmann R, Mann SG (2010). Oral ivermectin versus malathion lotion for difficult-to-treat head lice. N Engl J Med.

[16] Richard-Lenoble D, Chandenier J, Gaxotte P (2003). Ivermectin and filariasis. Fundam Clin Pharmacol.

[17] Kobylinski KC, Sylla M, Chapman PL, Sarr MD, Foy BD (2011). Ivermectin mass drug administration to humans disrupts malaria parasite transmission in Senegalese villages. Am J Trop Med Hyg.

[18] Chaccour C, Lines J, Whitty CJ (2010). Effect of ivermectin on *Anopheles gambiae* mosquitoes fed on humans: the potential of oral insecticides in malaria control. J Infect Dis.

[19] WHO. Notes on the design of bioequivalence study: Ivermectin. Geneva: World Health Organization; 2015. https://www.who.int/neglected_diseases/vector_ecology/vectorcontrol/Insecticides_IRS_22_September_2018.pdf?ua=1.

[20] Kobylinski KC, Deus KM, Butters MP, Hongyu T, Gray M, da Silva IM (2010). The effect of oral anthelmintics on the survivorship and re-feeding frequency of anthropophilic mosquito disease vectors. Acta Trop.

[21] Cully D (1994). Cloning of an avermectin-sensitive glutamate-gated chloride channel from *Caenorhabditis elegans*. Nature.

[22] Chaccour CJ, Hammann F, Alustiza M, Castejon S, Tarimo BB, Abizanda G (2017). Cytochrome P450/ABC transporter inhibition simultaneously enhances ivermectin pharmacokinetics in the mammal host and pharmacodynamics in *Anopheles gambiae*. Sci Rep..

[23] Balkew M, Ibrahim M, Koekemoer LL, Brooke BD, Engers H, Aseffa A (2010). Insecticide resistance in *Anopheles arabiensis* (Diptera: Culicidae) from villages in central, northern and south west Ethiopia and detection of kdr mutation. Parasit Vectors..

[24] WHO (2016). Test procedures for insecticide resistance monitoring in malaria vector mosquitoes.

[25] Pampiglione S, Majori G, Petrangeli G, Romi R (1985). Avermectins, MK-933 and MK-936, for mosquito control. Trans R Soc Trop Med Hyg.

[26] Derua YA, Kisinza WN, Simonsen PE (2015). Differential effect of human ivermectin treatment on blood feeding *Anopheles gambiae* and *Culex quinquefasciatus*. Parasit Vectors..

[27] Smit MR, Ochomo EO, Aljayyoussi G, Kwambai TK, Abong’o BO, Chen T (2018). Safety and mosquitocidal efficacy of high-dose ivermectin when co-administered with dihydroartemisinin-piperaquine in Kenyan adults with uncomplicated malaria (IVERMAL): a randomised, double-blind, placebo-controlled trial. Lancet Infect Dis..

[28] Foley D, Bryan J, Lawrence G (2000). The potential of ivermectin to control the malaria vector *Anopheles farauti*. Trans R Soc Trop Med Hyg.

[29] Fritz M, Siegert P, Walker E, Bayoh M, Vulule J, Miller J (2009). Toxicity of bloodmeals from ivermectin-treated cattle to *Anopheles gambiae s.l*. Ann Trop Med Parasitol..

[30] Alout H, Krajacich BJ, Meyers JI, Grubaugh ND, Brackney DE, Kobylinski KC (2014). Evaluation of ivermectin mass drug administration for malaria transmission control across different West African environments. Malar J..

[31] Gardner K, Meisch M, Meek C, Biven W (1993). Effects of ivermectin in canine blood on *Anopheles quadrimaculatus*, *Aedes albopictus* and *Culex salinarius*. J Am Mosq Control Assoc..

[32] Jones J, Meisch M, Meek C, Bivin W (1992). Lethal effects of ivermectin on *Anopheles quadrimaculatus*. J Am Mosq Control Assoc..

[33] Bockarie M, Hii J, Alexander N, Bockarie F, Dagoro H, Kazura J (1999). Mass treatment with ivermectin for filariasis control in Papua New Guinea: impact on mosquito survival. Med Vet Entomol.

[34] Smit MR, Ochomo EO, Waterhouse D, Kwambai TK, Abong’o BO, Bousema T (2019). Pharmacokinetics-pharmacodynamics of high-dose ivermectin with dihydroartemisinin–piperaquine on mosquitocidal activity and QT-prolongation (IVERMAL). Clin Pharmacol Ther.

[35] Sampaio VS, Beltrán TP, Kobylinski KC, Melo GC, Lima JB, Silva SG (2016). Filling gaps on ivermectin knowledge: effects on the survival and reproduction of *Anopheles aquasalis*, a Latin American malaria vector. Malar J..

[36] Chaccour C, Rabinovich NR (2017). Ivermectin to reduce malaria transmission II. Considerations regarding clinical development pathway. Malar J..

[37] Tesh RB, Guzman H (1990). Mortality and infertility in adult mosquitoes after the ingestion of blood containing ivermectin. Am J Trop Med Hyg.

